# Pain Management in Patients with Multiple Myeloma: An Update

**DOI:** 10.3390/cancers11122037

**Published:** 2019-12-17

**Authors:** Flaminia Coluzzi, Roman Rolke, Sebastiano Mercadante

**Affiliations:** 1 Department of Medical and Surgical Sciences and Biotechnologies, Sapienza University of Rome, Polo Pontino, 04100 Latina, Italy; 2 Department of Palliative Medicine, Medical Faculty RWTH Aachen University, 52062 Aachen, Germany; rrolke@ukaachen.de; 3 Main regional center for Pain Relief & Supportive Care, La Maddalena Cancer Center, 90100 Palermo, Italy; 03sebelle@gmail.com

**Keywords:** pain, multiple myeloma, neuropathic pain, skeletal-related events, bisphosphonate, denosumab, opioids, chemotherapy induced neuropathic pain, post-herpetic neuralgia, cancer survivors

## Abstract

Most patients with multiple myeloma (MM) suffer from chronic pain at every stage of the natural disease process. This review focuses on the most common causes of chronic pain in MM patients: (1) pain from myeloma bone disease (MBD); (2) chemotherapy-induced peripheral neuropathy as a possible consequence of proteasome inhibitor therapy (i.e., bortezomib-induced); (3) post-herpetic neuralgia as a possible complication of varicella zoster virus reactivation because of post-transplantation immunodepression; and (4) pain in cancer survivors, with increasing numbers due to the success of antiblastic treatments, which have significantly improved overall survival and quality of life. In this review, non-pain specialists will find an overview including a detailed description of physiopathological mechanisms underlying central sensitization and pain chronification in bone pain, the rationale for the correct use of analgesics and invasive techniques in different pain syndromes, and the most recent recommendations published on these topics. The ultimate target of this review was to underlie that different types of pain can be observed in MM patients, and highlight that only after an accurate pain assessment, clinical examination, and pain classification, can pain be safely and effectively addressed by selecting the right analgesic option for the right patient.

## 1. Introduction

Multiple myeloma (MM) is the second most common haematological malignance after non-Hodgkin lymphoma, representing 1.8% of all new cancer cases and the 14th cause of cancer death in the US [1]. Patients with MM may suffer from pain at different stages of their disease, with a prevalence of 73% and a strong negative impact on their quality of life (QoL) [2].

Osteolytic bone lesions are one of the most common complications of MM, with up to 90% of patients complaining of bone pain. In one third of patients, MM is diagnosed after a bone fracture, leading to severe pain and increased morbidity and mortality [3]. Due to the drugs used for treating MM, many patients suffer from chemotherapy-induced peripheral neuropathy (CIPN), which significantly affects quality of life. The proteasome inhibitor bortezomib has also been associated with CIPN in over 70% of treated patients [4]. Chemotherapy-induced immunosuppression makes patients more likely to develop infections, including reactivation of herpes zoster virus (HZV), which causes herpetic neuralgia, and post-herpetic neuralgia in a smaller percentage [5]. This is one of the most common forms of chronic localized neuropathic pain in MM patients.

Finally, the improvement in therapeutic options for MM, together with supportive care, have significantly increased the percentage of patients who survive the disease; however, they may present specific “cancer survivor” syndrome, characterized by chronic pain, fatigue, insomnia, depression, physical and psychological impairment, cognitive dysfunction, cancer-related neuropathies, and overall reduced quality of life [6].

In this narrative review, the authors intended to offer an overview of the different pain syndromes that physicians, particularly non-pain specialists, may encounter when they manage patients with MM. The pathophysiological mechanisms of chronic pain, along with the intensity, are the basis for establishment of an appropriate analgesic plan for adequate pain relief [7] and for improving QoL in MM patients.

## 2. Myeloma Bone Disease

Specific cancer types, including MM, lung, prostate, breast, and kidney cancer, are commonly associated with bone metastases. Bone lesions often lead to skeletal complications, which are commonly described as skeletal-related events (SREs), including pathological fracture, the need for bone radiotherapy or bone surgery, spinal cord compression, and hypercalcemia. These complications may reduce overall survival and are accompanied by severe chronic pain, loss of mobility and social role, and significant decrease in QoL [8].

Osteolytic bone lesions occur in 80%–90% of patients with MM, with back localization being a presenting feature in over three quarters of patients. Myeloma bone disease (MBD) is associated with a number of clinical consequences including bone pain (70%–80%), fractures (50%–60%), hypercalcemia (15%), and spinal cord compression (2%–3%) [9]. Pain associated with MBD and the resulting reduced mobility are significant risk factors for deep venous thrombosis, pulmonary complications, osteoporosis, and decubitus ulcers.

The European Society for Medical Oncology (ESMO) Clinical Practice Guidelines suggest a multidisciplinary approach, including all the health care professionals (HCPs) involved in the management of cancer patients [8]. Treatment strongly depends on the bone disease localization, as a single or multiple bone metastases. In general, all treatments for metastatic bone disease have a palliative purpose to reduce pain and improve functionality.

### 2.1. Pathophysiology of Bone Pain

The adult skeleton is innervated by thinly myelinated, tropomyosin receptor kinase A (TrkA)^+^ sensory nerve fibers (A-delta), while it does not receive innervation from larger A-beta fibers and TrkA^−^ unmyelinated C fibers. Bones are deep structures that do not require the sensitivity of the skin to fine touch, light pressure, and brushing; therefore, they do not receive A-beta fiber innervation. Bone sensory fibers are probably silent nociceptors, which are activated only in case of trauma or injury [10]. Acute and sharp pain after bone fracture is related to the sensory innervation of the periosteum, in which, conversely to the cortical bone, the density of A-delta and C-fibers is 1000 times greater [11]. Normal articular cartilage is completely free of any innervation by sensory nerve fibers, while synovial membranes and the subchondral bone are innervated and receive vascular supply. Bones also receive adrenergic and cholinergic sympathetic nerve fibers. Following bone injury, the sprouting of sympathetic nerves has been shown to accompany the ectopic sprouting of primary afferent sensory fibers; therefore, modulating sympathetic nerve sprouting may significantly decrease pain behavior in experimental models [12].

Investigating skeletal pain pathophysiology may suggest potential new targets of analgesic treatment. Future targets for analgesia in MM-induced bone pain could also include the acid-sensing channels 3 (ASIC3). Protons released during bone resorption create an acidic bone microenvironment that excites bone sensory neurons via activation of ASIC3 [13].

Bone injury, similarly to other musculoskeletal conditions, induces peripheral and central sensitization, which amplifies the perception of pain. In particular, the neuroplasticity phenomena occurring in the dorsal horn of the spinal cord causes exaggerated pain sensation after even non-painful stimuli (allodynia) or increased pain perception after even mild painful stimuli (hyperalgesia). These central nervous system functional and structural modifications are implicated in the process of pain chronification [14]. Although we do not have much information on the specific mechanisms by which skeletal pain induces chronification, we know that the skeleton is much more able than skin or muscle to induce central sensitization [10].

These finding suggest the use of a central analgesic for managing severe chronic bone pain, and the integration of adjuvants such as gabapentioids and antidepressants to manage the neuropathic component of MBD.

MBD is characterized by dysregulation of the physiological bone remodeling, defined as the interaction between bone marrow, osteoclast, osteoblasts, osteocytes, and the immune cells. MM cells alter the bone marrow microenvironment and induce apoptosis in osteocytes. Additionally, growth factors such as osteopontin, released by the resorptive process, increase the growth of MM cells [15] and inhibit cytotoxic T and NK cell action against MM cells [16]. Osteocytes and MM cells produce factors that inhibit osteoblastogenesis, such as DKK-1, sFRP-2, and sclerostin. The receptor activator of nuclear factor-kappa B (RANK) and its ligand (RANKL) pathway plays a key role in regulating osteoclast differentiation, while the wingless-type (Wnt) and beta-catenin pathway is the cardinal regulator of osteoblast activity. The net effect of this bone metabolism dysregulation is increased osteoclast function and impaired osteoblast activity, leading to reduced bone density and osteolysis [17].

### 2.2. Management of Myeloma Bone Disease

Management of MBD is always palliative, but it is essential for improving patients’ quality of life and includes antiresorptive therapies, with the aim of preventing further SREs, and radiotherapy, surgical procedures, corticosteroids, and analgesics in order to reduce the intensity of bone pain.

#### 2.2.1. Antiresorptive Therapy

ESMO practice guidelines on multiple myeloma strongly suggest the use of antiresorptive treatments in myeloma patients with bone disease for two years, and to eventually restart the treatment in relapsed patients [8]. Antiresorptive treatments include bisphosphonates and the recently approved denosumab.

##### Biphosphonates

Bisphosphonates (BPs) are the cornerstone of antiresorptive therapy in the management of MM. They act by inhibiting bone resorption, with a direct apoptotic effect on osteoclasts, which rapidly uptake BPs bound to the exposed mineralized bone [18]. Bone lesions often do not heal despite adequate anti-myeloma treatment, and are complicated by concomitant osteoporosis, leading to the recommendation for using BPs in any patient with BMD [19].

Oral clodronate (CLO), intravenous pamidronate (PAM), and intravenous zoledronic acid (ZA) have been approved for the management of MBD. Overall evidence is weak to recommend one bone-modifying agent over another and to define the optimal duration of the treatment. The main results of randomized controlled trials (RCTs) on BPs in MBD are presented in Table 1 [20,21,22,23,24,25,26].

The European Myeloma Network suggests all MM patients presenting with adequate renal function and osteolytic disease be treated with intravenous BPs [27]. Similarly, the American Society of Clinical Oncology (ASCO) guidelines suggest starting with an intravenous BP in any patient with myeloma and evidence of bone disease. Pamidronate 90 mg over at least 2 h or zoledronic acid 4 mg over at least 15 min every 3 to 4 weeks are recommended. Bone-modifying treatment should be continued for up to 2 years [28]. Two clinical trials evaluated the use of less-frequent dosing of zoledronic acid (every three months), compared with traditional once monthly administration, in order to reduce nephrotoxicity. Both studies showed that the incidence of SREs did not differ among the two treatment plans [21,29]. Renal toxicity and osteonecrosis of the jaw (ONJ) are the most critical side effects of BPs, and are related to the dose, duration of exposure, and plasmatic concentration.

A recent Cochrane meta-analysis evaluated the role of BPs in improving overall survival in MM by analyzing 24 placebo-controlled RCTs and four RCTs versus an active comparator. The results showed that BPs may reduce pathological vertebral fractures, SREs, and pain in MM patients; however, there is only moderate evidence for their reducing mortality [30].

In general, multiple RCTs on BPs in MM have shown a significant reduction in the incidence of SREs; however, data on their efficacy as analgesics are still lacking. The Medical Research Council (MRC) Myeloma IX trial reported a significant improvement in pain, fatigue, QoL, and physical functioning in MM patients treated with combined therapy with BPs (clodronic acid or zoledronic acid) and induction treatment [31].

##### Denosumab

In 2018, the Food and Drug Administration (FDA) approved denosumab for the prevention of SREs in patients with MM. Denosumab is a fully human monoclonal antibody that targets RANKL, which is an essential mediator for osteoclast survival and activation. In a recent phase 3, double-blind RCT, denosumab in patients with newly diagnosed MM was been shown to be non-inferior to zoledronic acid for time to first SRE, suggesting a potential role as an alternative to BPs in the management of MBD. Overall survival and side effects were similar in the two groups, with neutropenia being the most common (15% in both groups) and pneumonia being the most serious (8% in both groups) adverse events [26].

Denosumab is administered subcutaneously at 120 mg monthly. Of relevance could be the different impact of these two drugs on renal function, which is impaired in up to 60% of patients with MM, often limiting the use of BPs. Nephrotoxicity is, indeed, a well-known side effect of zoledronic acid, while denosumab is better tolerated in terms of significant increases of creatinine levels. No data were available in this trial on the analgesic effects of the two comparators.

The number of MM patients treated with denosumab is still too limited to suggest any specific recommendation on its use and indications on how to stop. Denosumab does not show prolonged activity after discontinuation, and therefore immediate bridging with BPs is recommended [27].

#### 2.2.2. Radiotherapy

Radiotherapy (RT) alone is generally highly effective for bone pain, with a response rate higher than 85% [32]. The response is usually fast, with about 50% of patients reporting pain relief within the first two weeks of treatment. This finding points towards a RT effect due rather to an immediate reduction of the inflammatory MM response in the bone marrow than to destruction of myeloma tissue. RT has been shown to reduce analgesic consumption, improve neurological symptoms, and promote physical function. In MM patients with localised disease, the response rate was 93% at 1 year and 82% at 2 years [33]. There is strong evidence nowadays supporting single-fraction radiotherapy (8 Gy in one fraction), because no differences have been detected when compared with fractionated therapy (30 Gy in 10 fractions) in MM patients in terms of pain relief [34]. Retreatment of recurrent bone pain should be offered with a further single dose of 8 Gy [35]. Palliative RT should be limited as much as possible to preserve residual bone marrow activity. Therefore, RT is the elective treatment for patients with localized disease, while patients with widespread lesions require additional treatment approaches including bone-target agents.

#### 2.2.3. Vertebroplasty/Kiphoplasty

Patients with pathological vertebral fractures and without spinal cord compression should be considered for vertebroplasty. Percutaneous vertebroplasty is a minimally invasive procedure involving the injection of polymethylmethacrylate (PMMA), bone cement, into the vertebral body. Kyphoplasty is a similar procedure where an inflatable balloon is placed into the vertebral body before the injection of bone cement. Both techniques are indicated in patients with vertebral compression fractures (VCFs) and result in effective pain relief, better mobility, and improved performance status [36]. These minimally invasive bone augmentation techniques are regarded as being very safe with a low incidence of possible complications, the most common being small cement leaks which, depending on the site of extravasation, may lead to nerve root compression, radiculopathy, peripheral disk irritation, and paravertebral tissue imbibition.

The analgesic effect of these techniques may be related to different factors. PMMA has a direct cytotoxic effect, which could cause necrosis of the tumor tissue, and has been shown to stabilize microfractures. Moreover, heat generated during the polymerization of PMMA may contribute to destruction of nerve endings in the vertebral body [37].

The Cancer Patient Fracture Evaluation (CAFE) study was a multicenter RCT which randomized patients who had cancer and one to three painful VCFs to receive kyphoplasty or non-surgical management. Back-specific functional status at one month was significantly improved in the kyphoplasty group, with the report of a rapid onset of pain relief [38]. However, the heterogeneity of study designs, outcomes, and populations still suggests that the current literature provides inconsistent data, and further studies should delineate confounding variables. Recommendations for performing these procedures are weak. Further studies are required to improve the strength of evidence available before recommending these procedures on large scale [39].

#### 2.2.4. Corticosteroids

Corticosteroids such as prednisone and dexamethasone are included in all major treatment regimens in MM, both in front-line treatment and for relapse/refractory disease [40]. Moreover, they are used as adjuvant therapy for painful bone metastases. According to a recent survey of palliative care providers in US, dexamethasone is the most commonly prescribed corticosteroid [41]. The rate of pain relief ranges between 30% and 70%, however their use is particularly useful for reducing pain flares during radiotherapy for patients with bone metastases [42]. High doses of corticosteroids may be administered in emergencies in patients where vertebral fractures cause spinal cord compression [43].

#### 2.2.5. Analgesics

Adequate pain control is of crucial importance for the quality of life of myeloma patients, and analgesics are essential in the management of BMD. Osteolytic lesions are often the first clinical symptom of MM. Even in patients where invasive treatments (i.e., vertebroplasty) are indicated, an effective analgesic treatment should be promptly started while waiting for the surgery. Moreover, most patients treated with other systemic treatments, such as bone-target agents, may require analgesic support to obtain adequate pain relief.

Managing chronic pain require adequate pain assessment through accurate medical history, physical examination, review of clinical records, and further investigations. Pain should be evaluated in terms of location, onset, duration, severity, and quality. Alleviating and aggravating factors should be investigated. Impact of pain on mood, sleep, daily activities, functionality, and QoL is essential for assessment. Previous analgesics, reported adverse events, and ongoing response to treatment is a useful basis for managing pharmacological treatment. Personal characteristics, comorbidities, and concomitant medications help physicians to select the right drug for the right patient [44].

Among non-opioids, non-steroid anti-inflammatory drugs (NSAIDs) are not recommended due to their potential nephrotoxicity, especially in MM patients where kidney function is usually affected by the disease itself and by BPs. Acetaminophen, up to 4 g daily, is a safe and well tolerated drug for mild acute and chronic pain, even in the most vulnerable patients. There is no evidence to support or refuse the use of NSAIDs or acetaminophen alone or in combination with opioids for mild to moderate cancer pain [45].

Opioids are still the mainstay in the management of severe cancer pain. The oral route of administration should be considered as the first choice, except when oral intake is not possible. Individual titration is strongly recommended to achieve adequate analgesia without intolerable side effects. Both short-acting opioids (SAO) and long-acting opioids (LAO) can be used during titration, while transdermal formulations should be reserved for patients using a stable dose of opioid analgesics [45].

Opioids act mainly in the central nervous system, where they modulate the ascending pain pathway. Opioids induce analgesia by binding to mu-opioid receptors (MOR) and modulating ion channel performance. They reduce pain transmission by inhibiting voltage-gated calcium channel influx in the pre-synaptic neuron (primary afferent fibre), and subsequent inhibition of the neurotransmitter release. Secondly, post-synaptically, they increase potassium conductance of the second-order sensory neuron in the spinal cord dorsal horn [46]. They are the most potent analgesic drugs; however, their use requires caution, because of the potential side effects associated with their use, particularly constipation and other adverse gastrointestinal events. MOR are located peripherally in the myoenteric and submucosal plexuses in the gastrointestinal tract, by which endorphins physiologically modulate peristalsis, while exogenous opioids cause opioid-induced bowel dysfunction (OIBD) [47]. Peripherally acting MOR antagonists (PAMORAs) can be used for managing OIBD without affecting the central analgesic effects of opioids [48].

The analgesic effect of opioids is more evident for the nociceptive component of pain, while adjuvants may be required to manage neuropathic pain. Neuropathic pain characteristics are detected in 20% of patients with cancer [49]; however, this percentage rises to 40% when mixed pain is included and to about 70% in patients with bone metastases [50]. There are no data to support any one opioid over another; however, one could argue that atypical opioids, with a lower activity on MOR, could be safely used to reduce the risk of opioid-related adverse events and to target a mixed-pain situation in cancer-related pain, where nociceptive and neuropathic mechanisms are in action at the same time [51].

Tapentadol, due to its dual mechanism of action (MOR agonist and noradrenaline reuptake inhibitor), has been effectively used in MM patients for the management of moderate to severe pain caused by MBD, and has been shown to be well tolerated and effective for reducing the neuropathic component of pain [50]. Its activity on the descending inhibitory pathway, due to the increased bioavailability of noradrenaline in the synaptic cleft, has been shown to be the main determinant of its analgesic activity in animal models of neuropathic pain [52].

In MM patients, renal function may be a limiting factor in the prescription of some opioids such as codeine and morphine, which may accumulate in patients with moderate to severe renal insufficiency and may have toxic side effects. Oxycodone and hydromorphone require dosage adaptation, while buprenorphine and fentanyl are considered the first-choice opioid drugs in patients with severe kidney failure (creatinine clearance < 30 mL/min). Tapentadol does not require dosage adaptation for mild to moderate renal failure, but data on its use in end-stage renal disease are not available [53].

Another emerging concern about chronic opioid use, particularly in MM patients, is the possible negative impact of MOR agonists on the bone density. Opioids may affect bone density via direct activity on osteoblasts or via indirect activity on the hypothalamic–pituitary–adrenal (HPA) axis [54]. Opioids are well known to cause opioid-induced androgen deficiency (OPIAD), particularly in men using daily doses higher than 100 mg oral equivalent of morphine. One of the possible consequence of androgen deficiency is impaired bone mass density, as detected by dual-energy X-ray absorptiometry (DEXA) scan. The first observations were recorded in opioid abusers using very high doses of opioids, and in patients under methadone maintenance treatment [55]. Strong analgesics with a reduced activity on MOR have been shown to have a lower impact on the endocrine system of chronic pain patients [56].

The pharmacokinetic properties of opioids are essential for tailoring analgesia in poly-medicated patients [57]. For example, the frequently used in MM proteasome inhibitor bortezomib is extensively metabolized by the hepatic cytochrome P450 (CYP) isoenzymes CYP3A4 and CYP2C19. Co-administration of other drugs, including analgesics, metabolized by the same system, may result in drug–drug pharmacological interactions. In this context, at least the use of the co-analgesic dexamethasone, which is the most commonly used steroid in spinal cord compression and is a weak CYP3A4 inducer, does not interfere with MM treatment, as its administration did not affect the exposure to bortezomib in MM patients [58]. Regarding opioids, CYP3A4 is involved in the metabolism of oxycodone (N-demethylation to inactive noroxycodone) [59] and fentanyl. Fentanyl may inhibit the activity of CYP3A4, resulting in increased bioavailability and toxicity of chemotherapeutic agents, e.g., paclitaxel [60]. The same effect could be potentially observed in MM patients using bortezomib; however, clinical data on pharmacological interactions between opioids and bortezomib are not available to date. Caution should be taken when prescribing CYP3A4 metabolized opioids to MM patients. Morphine, hydromorphone, and tapentadol are the currently available opioids without or with minimal interference with CYP450.

#### 2.2.6. Management of Breakthrough Cancer Pain

From a clinical perspective, the main problem with opioid analgesia in the presence of vertebral involvement, is that pain at rest may be easily controllable with analgesics, while pain on movement may be severe enough to limit physical activity. This type of predictable event represents a subset of a large phenomenon commonly called breakthrough cancer pain (BTcP) [61]. BTcP is, by definition, a transitory increase in pain to greater than moderate intensity which occurs on top of a stable background pain, otherwise well-controlled with stable opioid doses given around the clock [62]. While an increase in dose of the selected opioid used for background pain may provide more analgesia allowing more chance of movement and fewer BTcP episodes induced by movement, this may result in the development of adverse effects. Thus, it is necessary to find an individual compromise, according to the level of quality of life of the patient, to achieve the optimal analgesia with limited adverse effects. In some cases, opioid switching may provide an enlargement of the therapeutic window. This approach should be tailored to the individual characteristics of the patient. On the other hand, this strategy cannot solve the problem in all the cases. Again, the number of episodes evoked by movement should be balanced with the level of activity of the patient. Some of these episodes could be preceded by analgesics given before starting an expected painful activity, for example giving immediate-release oral morphine 30 min before, or rapid onset fentanyl products just 5–10 min before. Once the episode of high intensity occurs, it is necessary to provide a rapid analgesia, ideally with drugs with a rapid onset like fentanyl preparations. On the other hand, patients may spontaneously stop their activity as a spontaneous reaction against a physical insult [63]. Rapid-onset opioids (ROO) have been used for managing BTcP in MM patients with VCFs [64].

General rules for the use of ROO in the management of BTcP include: (a) patients with chronic background pain (≥12 h/day during previous week), which is adequately controlled using a stable daily dose of LAO ≥ 60 mg equivalent of morphine; (b) maximum 4 episodes of BTcP per day with an intensity ≥7 on a 11 point numerical rating scale; and (c) dose selection by using titration for patients using low doses of baseline opioids and dose proportionality (1:6) for high-dose patients [65].

## 3. Chemotherapy-Induced Peripheral Neuropathy (CIPN)

The abovementioned chemotherapeutic agent bortezomib is a well-established drug for the treatment of multiple myeloma and mantle cell lymphoma. Among other side effects, bortezomib can induce a mild to moderate dose-dependent sensory neuropathy in MM patients [66,67]. Risk factors include the bortezomib dose and a preexisting neuropathy [68]. Besides bortezomib, other immunomodulatory agents addressing MM, such as thalidomide and lenalidomide, or chemotherapies including vinca alkaloids and cisplatin may contribute to this peripheral neuropathy [69]. Moreover, combination therapies of, for example, bortezomib and thalidomide may result in higher neuropathy rates [70].

### 3.1. Clinical Features

Bortezomib-induced peripheral neuropathy (BIPN) is clinically characterized by neuropathic pain in fingers and toes, paraesthesias, burning dysaesthesias, numbness, and other clinical features such as (rare) sensory ataxia [67]. Median pain intensity and the McGill pain questionnaire sum score point towards a moderate to severe neuropathic pain within BIPN. In a recent study, up to 77% of patients with MM had a mean pain intensity > 4 (numerical rating scale; 0 = “no pain” to 10 = “most intense pain imaginable”) [66]. Grade 1 or 2 BIPN can occur in up to 75%, and Grade 3 or 4 BIPN in 30% of newly diagnosed MM patients [68]. Other studies have reported a lower prevalence with overall BIPN incidence rates of 35% in myeloma patients, which spontaneously resolved in about 88% of those patients [71]. Others have reported lower frequencies of complete spontaneous restauration of nerve fiber function or improvement of BIPN in at least 71% of myeloma patients [72]. It is important to note that MM itself can increase the risk for developing peripheral neuropathy with or without pain [69].

Clinical electrophysiology has shown axonal rather than demyelinating sensory neuropathy in BIPN [66] with abnormal sural nerve neurography in 86% of patients with MM. Increased thermal and mechanical detection thresholds derived from quantitative sensory testing reflect the dysfunction of A-beta-, A-delta-, and C-fibers [68]. Histological findings of early BIPN stages demonstrate axonal swelling rather than a diminished intraepidermal nerve fiber density in skin biopsies [66]. Thalidomide and bortezomib combination therapies can lead to a more prominent, reversible, length-dependent, sensory rather than motor, and predominately axonal, large-fiber more than small-fiber polyneuropathy [70].

### 3.2. Mechanisms of Action of Immunomodulatory and Chemotherapeutic Agents

Bortezomib is a proteasome inhibitor affecting the development of the cytoskeleton and many other aspects of cell metabolism. The details of the mechanisms resulting in BIPN are still unknown. Several studies have suggested multifactorial pathways towards nerve fiber degeneration. Bortezomib induces aerobic glycolysis in sensory neurons, which may lead to the extrusion of metabolites sensitizing primary sensory afferents, finally causing pain [73]. One study in mice demonstrated bortezomib effects resulting in oxidative stress, mitochondrial dysfunction, cell apoptosis, and endoplasmatic reticulum stress [74]. Thalidomide is a glutamic acid derivate introduced in 1957 that was withdrawn from the market due to teratogenic side effects. It became available in Europe again in 2008 due to its diverse mechanisms of action against, for example, MM including anti-angiogenic, anti-inflammatory, and pro-apoptotic properties [69]. Lenalidomide is the 4-amino-glutamyl analogue of thalidomide with less neurotoxic side effects. Its anti-inflammatory potency is mediated through the downregulation of pro-inflammatory cytokines and upregulation of anti-inflammatory cytokines, and the reduction of cell surface adhesion molecules [75,76]. Cisplatin acts, amongst other effects, via the induction of mitochondrial stress and dysfunction [77]. Vinca alkaloids belong to the group of microtubule-targeting agents affecting cell proliferation [78].

### 3.3. Managing Chemotherapy-Induced Neuropathic Pain (CINP)

Chemotherapy-induced neuropathic pain should be treated like any other type of cancer-related neuropathic pain. This includes a systemic medical management focusing on tricyclic antidepressants, the serotonin/norepinephrine reuptake inhibitor duloxetine, or gabapentinoids. Focal pain spots can be treated topically with lidocaine or high-dose capsaicin patches [45]. Interestingly, complementary therapies such as acupuncture have shown a significant reduction of neuropathic pain, but also motoric impairment in BIPN Grade 2 or above [79]. In cases of relevant neuropathy, BIPN pain might be addressed by reducing subsequent bortezomib doses. In a non-randomized, observational trial in frail myeloma patients, low-dose subcutaneous bortezomib with oral prednisolone showed a low neuropathy rate of about 4% [80]. In general, dose reduction of bortezomib, thalidomide, lenalidomide, and cisplatin or vinca alkaloids needs to be balanced, reflecting the life-prolonging effects of such substances. It is significant that to date, no treatment exists for the prevention and treatment of peripheral neuropathy. Treatment is focused on symptom control with regard to the pain experience. Today, this type of neuropathy is spontaneously resolving in many cases after discontinuation of the immunomodulating or chemotherapeutic agents. The development of preventive strategies is a focus of current CIPN research.

## 4. Herpetic and Post-Herpetic Neuralgia

Infections are a leading cause of morbidity in MM patients, because of the immunosuppression related to anti-myeloma therapies (i.e., post-transplantation) and to the disease itself. The introduction of novel therapies such as bortezomib, thalidomide, and lenalidomide has improved the outcomes of MM patients, but it has probably increased the incidence and the spectrum of infections [81]. Proteasome inhibitors, especially bortezomib, have been shown to increase the risk of reactivation of varicella zoster viruses (VZV) in patients not receiving prophylactic anti-viral agents [82]. Reactivation usually occurs late during treatment [83]. Herpes zoster infection has been shown to be the most common cutaneous comorbidity in MM patients [84]. Zoster prophylaxis is indeed recommended in guidelines for MM patients receiving protease inhibitors. Patients taking bortezomib should be monitored for viral reactivation [85].

Post-herpetic neuralgia (PHN) is a potential complication after acute herpes zoster or VZV reactivation and herpetic neuralgia. PHN is a typical example of localized neuropathic pain (LNP), which is usually limited to a superficial, circumscribed, and easily identifiable area. LNP always reflects a peripheral nerve lesions which is dermatomal in distribution, does not cross the midline, and can be associated with positive sensory phenomena such as spontaneous burning pain, allodynia, and hyperalgesia [86].

In these conditions, topical treatments, including lidocaine 5% plasters and capsaicin 8% patches may be appropriate [87]. Topical treatments offer the advantage of a localized effect in the absence of systemic side-effects, i.e., related to the central nervous system. Their analgesic efficacy is not due to their systemic absorption, which is generally neither required nor desired in these formulations. In cancer patients, where organ failures (kidney or liver impairment) may limit the use of systemic drugs, their use is particularly advantageous. However, data on LNP in MM patients are still lacking [88].

Lidocaine 5% plaster is a first-choice drug in PHN, as a single therapy or as add-on therapy together with systemic analgesics, particularly adjuvants such as gabapentinoids and antidepressants. Lidocaine 5% shows good short- and long-term tolerability, with the most common adverse events being application site reactions [89]. Superficial and localized pain presenting with positive phenomena such as allodynia and hyperalgesia is a strong outcome predictor of success. Conversely, patients suffering from widespread, deep, long-term pain, with areas of anesthesia, are likely not to have benefits from topical lidocaine [90].

Capsaicin is a highly selective agonist of the transient receptor potential vanilloid-1 (TRPV-1) receptor. Capsaicin 8% dermal patches are indicated for the treatment of peripheral neuropathic pain in adults either alone or in combination with other analgesics. A single 60 min application of capsaicin 8% provides prolonged pain relief in patients with PHN, with good tolerability. Transient application-site reactions such as reddening of the skin are the most common adverse events [91].

Current guidelines on neuropathic pain suggest as first-line treatment, in monotherapy, gabapentin (1200–3600 mg daily), pregabalin (300–600 mg daily), duloxetine (60–120 mg daily), venlafaxine (150–225 mg daily), and tricyclic antidepressants (TCAs) (25–150 mg daily). When these drugs do not provide adequate analgesia, weak opioids such as tramadol are recommended. Strong opioids, despite their number needed to treat (NNT) which offer significantly better results than other systemic drugs, are not recommended in PHN because their efficacy in neuropathic pain is still under discussion, and also largely because of their potential risk of abuse, which led the US into its recent opioid crisis [92]. Atypical opioids such as tapentadol, due to its dual mechanism of action, could have a role in cancer patients with neuropathic pain [93]. In 2012, tapentadol was the first opioid approved by the FDA for neuropathic pain associated with diabetic peripheral neuropathy, however, studies on PHN are not available yet. There is insufficient evidence to support or refute the use of other opioids, such as oxycodone, buprenorphine, and tramadol, which have been shown to provide clinically relevant pain relief in highly selected patients with neuropathic pain syndromes [94].

Unfortunately, RCTs for the management of cancer-related neuropathic pain are sparse, and most recommendations are extrapolated from studies on chronic non-cancer neuropathic pain syndromes. Dealing with the task of limited evidence in this context, the ESMO has recently published a cancer pain practice guideline which also addreses the management of cancer-related neuropathic pain [45].

## 5. Pain in Cancer Survivors

Since the introduction of bortezomib, thalidomide, and lenalidomide, median overall survival in patients with MM younger than 50 years was calculated to be >10 years in 2014. Since the introduction of newer drugs and combination therapies, about 70% of patients with MM now achieve complete responses and minimal residual disease (MRD) negativity [95].

As survival time has increased with two-, three-, and four-drug combination regimens, the number of cancer survivors presenting with chronic pain has increased as well. The concept of being a “cancer survivor” is relatively new, without a consensus on its definition, but it is usually related to a patient who lives with and beyond cancer [96].

Despite few specific data being available on patients with MM who have experienced prolonged survival [97], it is arguable that they could suffer from chronic pain, as up to 40% of cancer survivors report having chronic pain [44]. Pain is the second most reported symptom in cancer survivors, after fatigue [98].

Apart from the above-described pain syndromes, cancer survivors may present a number of long-term symptoms, including fatigue, which is a persistent feeling of physical, emotional, or mental tiredness or exhaustion. Obstacles in treating chronic pain in these patients could be related to emotional difficulties, such as fear of recurrence, depression, anxiety, anger, and guilt. The bio-psychosocial model of chronic pain should be considered when managing cancer patients [99].

A survey conducted on 199 cancer survivors, and examining the differences between different types of cancer, showed that MM is associated with the highest percentage (33%) of patients suffering from chronic pain, compared with the other cancer types (breast, lung, colorectal, and prostate). Moreover, women experience more pain than men, and black people experienced more disability than white. Pain was closely related to depressive symptoms and poor functioning [100]

Fatigue and pain have been identified as the two most important factors affecting quality of life in long-term MM survivors, even in stable-phase disease. About 70% of these patients were prescribed analgesics and 68% of these were on opioids. Over 50% of survivors reported neuropathic pain and were managed mainly with gabapentinoids [101].

According to a recent survey among long-term survivors (5 years) after autologous hematopoietic cell transplantation (AHCT), 33% of MM patients reported use of medications for pain management. Worse physical and mental functioning were associated with pain and depression, which strongly affected quality of life [102].

Graft-versus-host disease (GvHD) is a potential consequence after allogeneic hematopoietic stem cell transplantation, which occurs in 20%–50% of transplant survivors. Neuromuscular manifestations of GvHD are rare, but may cause painful conditions and have a major impact on the QoL. Muscle cramps, immune-mediated neuropathies, and myositis have been described among peripheral nervous system manifestations of GvHD [103]. Patients affected from chronic GvHD have been shown to be more likely to receive prescription medications for pain, depression, and anxiety [104]. Active chronic GvHD has been shown to have a significant impact on all SF-36 components of QoL (physical, mental, and social) and on pain intensity, while resolved previous chronic GvHD does not impair QoL [105].

Exercise training is recommended in cancer survivors, while inactivity should be avoided. Exercise is a potent stimulus to endorphine production; therefore, it could improve not only mobility, but also analgesia, and probably mood [106].

Cancer survivors should be considered vulnerable patients, particularly for mood disorders. In these subjects, prescribing long-term opioid therapy can be challenging and require continued medical education, with specific familiarity with risk-mitigation tools and current guidelines, in light of the increasing fear about opioid abuse potential [107].

## 6. Conclusions

**Figure 1:**
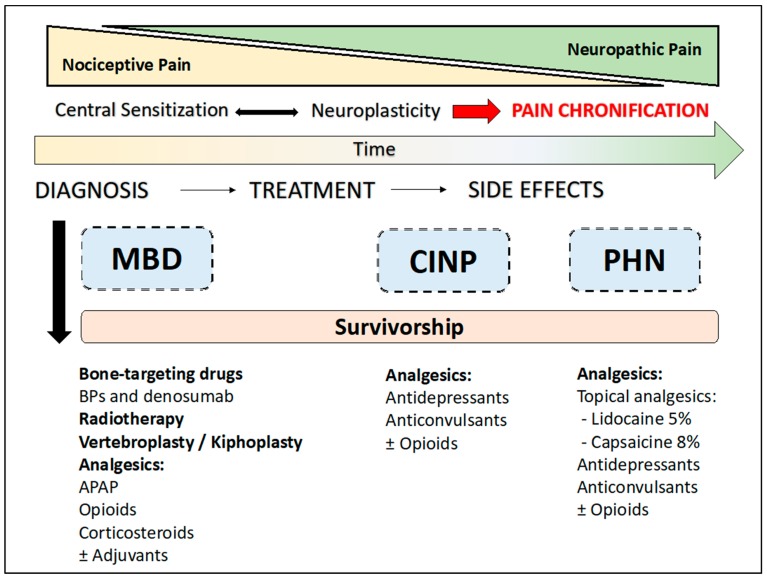
Pain management in multiple myeloma patients. APAP: acetaminophen (paracetamol); BPs: bisphosphonates; CINP: chemotherapy induced peripheral neuropathy; MBD: myeloma bone disease; PHN: post-herpetic neuralgia

Patients affected by MM suffer from chronic, moderate-to-severe pain at every stage of the natural disease process. Pain intensity is only one of the pain characteristics affecting the type of analgesia that should be selected. Adequate pain management requires identification of the pathophysiological mechanisms at the basis of chronic pain, and classification of pain as mainly nociceptive, mainly neuropathic, or mixed pain syndromes [98]. Central sensitization is known to play a key role in the transition from physiological adaptive pain to pathological maladaptive pain [108]. During MM progression, most possible emerging painful conditions will present with a progressively increasing neuropathic pain component, leading to a switch of the recommended first-line analgesic drugs (Figure 1). If in nociceptive pain conditions opioids still represent the cornerstone for managing moderate-to-severe pain, in neuropathic pain syndromes, antidepressants and anticonvulsants are the first-line analgesic treatments and opioids work only as “co-analgesics”, when analgesia is not satisfactory with the other abovementioned drugs. The role of topical analgesics for localized neuropathic pain is becoming more evident. Overall, long-term chronic pain is more likely to be neuropathic. Cancer-related neuropathic pain is very common in MM patients because of chemotherapeutic agents’ toxicity and possible complications of cancer-induced immunosuppression, such as infections. Physicians involved in the management of MM patients should be aware of the complexity of pain syndromes they may encounter in their clinical practice. Adequate education of non-pain specialists will help MM patients to receive adequate and early treatment for their chronic pain.

## Figures and Tables

**Table 1:** Main randomized controlled trials (RCTs) on bone-targeting agents in the management of myeloma bone disease (MBD).

Study	Treatment Drug	Treatment Dosing Schedule	Patients (n)	SRE Incidence (%)	Median Time to First SRE (mo)	Renal Toxicity (%)	Pain Reduction
[20]	PAM vs. Placebo	90 mg PAM every 4 wk for 9 cycles vs. Placebo	196 vs. 181	24 vs. 41 ^#^	Shorter in the placebo group ^#^	NA	PAM: significant decrease in bone pain scores °
[21]	ZA vs. PAM	4 or 8 mg i.v. ZA every 3–4 wk for 12 mo vs. 90 mg iv PAM every 3–4 wk for 12 mo	129 vs. 65	NA	12.5 vs. 9.4	NA	Pain reduction in both groups (no significant difference between groups)
[22]	PAM	30 mg PAM vs. 90 mg PMA	252 vs. 252	33.7 vs. 35.2	10.2 vs. 9.2	NA	Pain reduction in both groups (no significant difference between groups)
[23,24]	ZA vs. CLO	4 mg iv ZA every 3–4 wk vs. 1600 mg daily oral CLO	981 vs. 979	27 vs. 35 ^#^	NA	Similar for both groups	NA
[25]	ZA	ZA every 12 wk vs. ZA every 4 wk	139 vs. 139	55 vs. 60	NA	NA	No significant difference between groups at BPI scores
[26]	Denosumab vs. ZA	120 mg sc denosumab + i.v. placebo vs. i.v. 4 mg ZA + sc placebo every 4 weeks	859 vs. 859	43.8 vs. 44.6	22.8 vs. 24	10 vs. 17.1	NA

Modified from Terpos 2019 [17]. MBD: myeloma bone disease; PAM: pamidronate; ZA: zoledronic acid; CLO: clodronate; wk: weeks; mo: months; i.v.: intravenous; sc: subcutaneous; NA: not available; BPI: Brief Pain Inventory; ° *p* ≤ 0.05; ^#^ *P* ≤ 0.001.
